# Ileal malignant melanoma presenting with severe iron-deficiency anemia and active small-bowel bleeding: primary intestinal melanoma versus metastasis - a case report and narrative review

**DOI:** 10.3389/fonc.2026.1825602

**Published:** 2026-07-15

**Authors:** Pierfrancesco Di Cello, Paolo Izzo, Piero Luigi Alò, Rosalba Cipriani, Luciano Izzo, Claudia De Intinis, Silvia Lai, Marcello Molle, Daniela Messineo, Sara Izzo

**Affiliations:** 1 General Surgery Unit, Frosinone-Alatri Hospital Network (San Benedetto Hospital), Alatri, FR, Italy; 2 ”Pietro Valdoni” Department of Surgery, Policlinico Umberto I, Sapienza University of Rome, Rome, Italy; 3 Pathology Unit, “F. Spaziani” Hospital, Frosinone, Italy; 4 Internal Medicine Unit, San Benedetto Hospital, Alatri, FR, Italy; 5 Department of Translational and Precision Medicine, Nephrology Unit, Sapienza University of Rome, Rome, Italy; 6 Plastic Surgery Unit, Multidisciplinary Department of Medical-Surgical and Dental Specialties, University of Campania “Luigi Vanvitelli”, Naples, Italy; 7 Department of Radiological Sciences, Oncology and Pathology, Sapienza University of Rome, Rome, Italy

**Keywords:** gastrointestinal bleeding, ileum, iron-deficiency anemia, melanoma, metastasis, multiphasic computed tomography, PET/CT, primary gastrointestinal melanoma

## Abstract

**Background:**

Melanoma involving the small bowel is most often metastatic from an occult or regressed cutaneous primary; true primary small-bowel melanoma remains exceptional and controversial. A rigorous diagnostic work-up integrating cross-sectional imaging, whole-body metabolic staging, and detailed pathology is therefore mandatory.

**Case presentation:**

We report a 77-year-old man who presented with recurrent melena and severe iron-deficiency anemia (hemoglobin 6.8 g/dL) requiring transfusion. Multiphasic contrast-enhanced abdominal computed tomography (CT) demonstrated a hypervascular polypoid mass in the proximal ileum with signs of active intraluminal hemorrhage. The patient underwent an exploratory laparoscopy and segmental resection of approximately 20 cm of proximal ileum, including the adjacent mesentery. Histopathology showed malignant melanoma with transmural invasion and negative margins; regional lymph nodes were negative. Postoperative staging with contrast-enhanced chest CT, whole-body ^18F-FDG PET/CT, and contrast-enhanced brain MRI showed no residual or distant disease. Comprehensive dermatologic and ophthalmologic examinations did not reveal a cutaneous or ocular primary melanoma. At 24 months, the patient remains alive with no evidence of disease.

**Conclusions:**

This case underscores the value of multiphasic CT in identifying a bleeding small-bowel tumor and of ^18F-FDG PET/CT in confirming the absence of disseminated disease. Although an occult regressed primary can never be excluded with absolute certainty, the solitary ileal lesion, negative staging, and sustained disease-free follow-up support a diagnosis of probable primary ileal melanoma. We also provide an updated narrative review focusing on diagnostic criteria, imaging patterns, and practical elements that may strengthen classification.

## Background and introduction

Melanoma is predominantly a cutaneous malignancy; noncutaneous primaries (including ocular and mucosal disease) represent a small minority in large registries (1), with only a few dozen cases reported in the literature (2). Within the gastrointestinal (GI) tract, melanoma is classically considered metastatic until proven otherwise. Indeed, melanoma has long been recognized as a common source of GI metastases, with autopsy data suggesting that metastases may be present in up to 60% of patients, and the small bowel representing a frequent site of GI involvement (3). Primary melanoma of the small bowel is therefore an uncommon and debated entity. Most authors agree that when melanoma is found in the intestine, the critical diagnostic challenge is distinguishing a true mucosal primary from metastatic disease arising from an occult, regressed, or clinically overlooked primary melanoma elsewhere (4–6). This distinction has implications for staging, prognosis, and therapeutic planning, and it requires a multidisciplinary approach integrating clinical evaluation, imaging, endoscopic assessment when feasible, and pathology (7). From the radiologist’s perspective, prompt recognition of a bleeding or obstructing small-bowel mass, along with comprehensive staging to identify additional disease, is a central step. In this article, we present a surgically treated, node-negative ileal melanoma discovered in the setting of active small-bowel bleeding, and we review updated evidence regarding diagnostic criteria and imaging findings supporting the primary-versus-metastatic dilemma.

## Case presentation

A 77-year-old man presented to Alatri Hospital emergency department with recurrent melena and severe iron-deficiency anemia. Initial laboratory tests showed hemoglobin 6.8 g/dL, and a packed red blood cell transfusion was required. A multiphasic contrast-enhanced abdominal CT (arterial and portal venous phases) was performed as part of the diagnostic work-up. CT demonstrated a suspicious hyperenhancing lesion in the proximal ileum, approximately 80 cm from the ileocecal valve (**Figures 1A–C**). The mass measured 4.1 × 3.4 × 3.0 cm and appeared as a broad-based polypoid endoluminal lesion with an exophytic mural component. There was focal asymmetric mural thickening with partial luminal stenosis. Enhancement was marked in the arterial phase with internal heterogeneity. Hyperdense intraluminal material was present, consistent with recent intraluminal hemorrhage/contrast extravasation. Only a few subcentimeter mesenteric lymph nodes were noted, without unequivocally pathologic features, and no hepatic or peritoneal metastases were identified. After multidisciplinary discussion and agreement with the patient’s family, an exploratory laparoscopy was performed, followed by segmental resection of approximately 20 cm of proximal ileum and the adjacent mesentery, including palpable lymph nodes, with primary anastomosis. Gross examination revealed an ulcerated polypoid neoplasm measuring approximately 4 cm, predominantly endoluminal with infiltration of the bowel wall. Histology demonstrated a neoplastic proliferation composed of atypical epithelioid cells with prominent nucleoli and focal melanin pigment. Tumor infiltration was transmural, reaching the serosa. Surgical margins were free of tumor, and all resected lymph nodes were negative for metastasis. Immunohistochemistry showed diffuse positivity for S100 and SOX10, and positivity for HMB45 and Melan-A (MART-1). Cytokeratin AE1/AE3, CD117 (c-KIT), and DOG1 were negative. The Ki-67 proliferation index was approximately 30%. Junctional activity/an *in situ* component was not identified. Overall findings were diagnostic of small-bowel malignant melanoma (**Figures 2A–C**).

**Figure 1:**
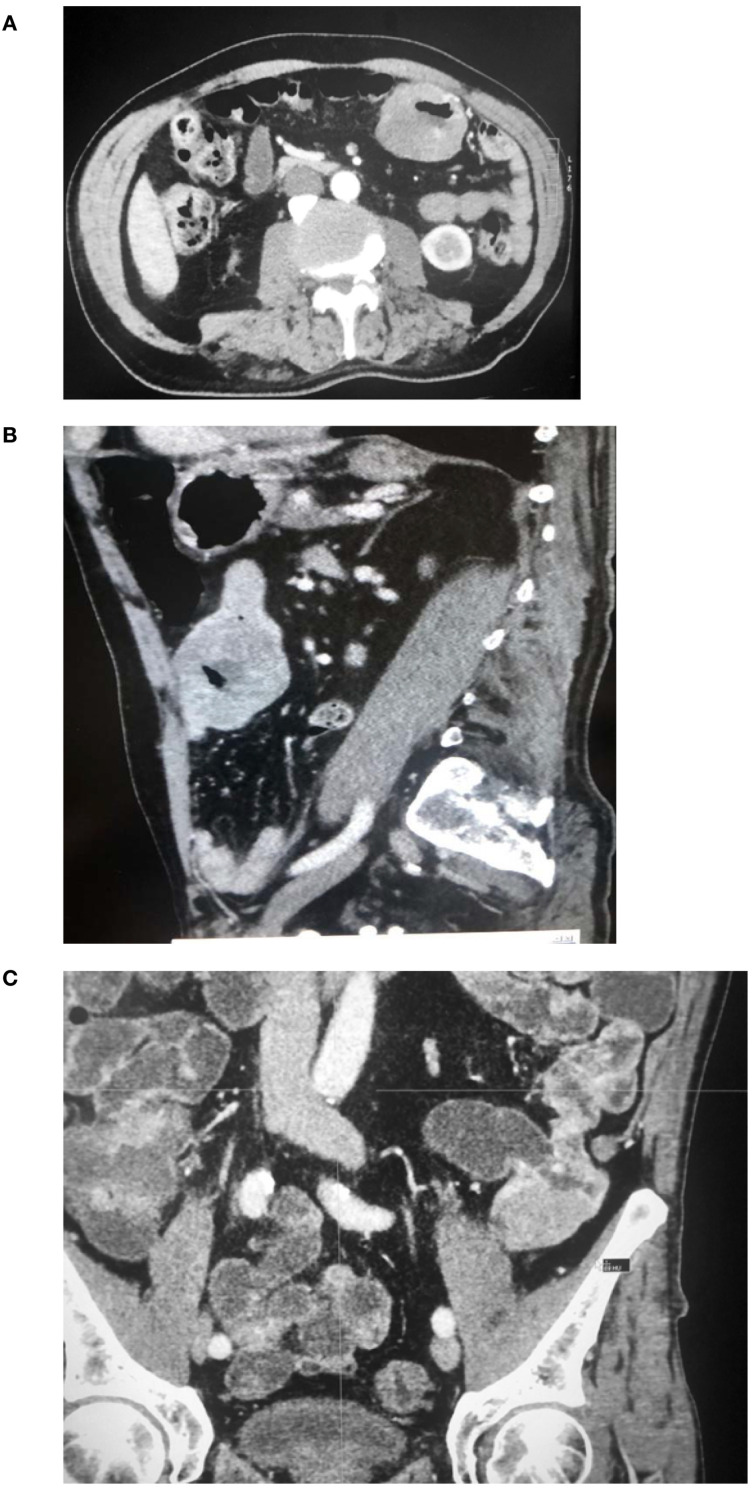
A CT axial plane, and B CT MPR sagittal plane. Multiphasic contrast-enhanced abdominal CT demonstrating a hypervascular broad-based polypoid endoluminal mass in the proximal ileum (4.1 × 3.4 × 3.0 cm), approximately 80 cm from the ileocecal valve, with focal asymmetric mural thickening and partial luminal narrowing. Marked arterial-phase hyperenhancement and intraluminal hyperdense material are consistent with active/recent intraluminal bleeding. **(A)** Axial image. **(B)** Sagittal multiplanar reconstruction.

**Figure 2:**
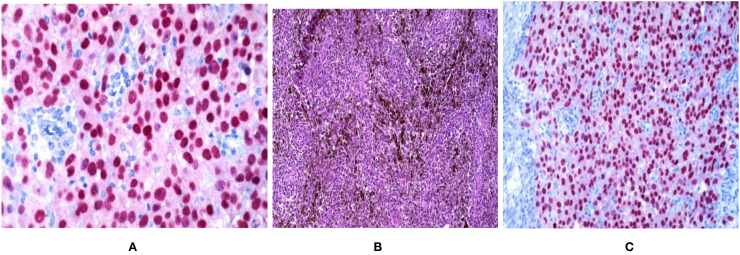
**(A–C)** Representative histology and immunohistochemistry of the ileal tumor showing an epithelioid malignant melanoma with focal melanin pigment and transmural invasion. Immunohistochemistry is positive for melanocytic markers (S100, SOX10, HMB45, and Melan-A) and negative for epithelial (AE1/AE3) and GIST markers (CD117 and DOG1).

Postoperative staging included contrast-enhanced chest CT, whole-body ^18F-FDG PET/CT, and contrast-enhanced brain MRI, all of which were negative for residual or distant disease. Comprehensive dermatologic and ophthalmologic examinations did not identify a primary cutaneous or ocular melanoma.

The case was discussed in a multidisciplinary tumor board. Given the absence of residual disease, absence of metastases, and negative lymph nodes, active surveillance without adjuvant immunotherapy was selected.

Follow-up consisted of clinical evaluation and laboratory testing every 6 months, annual contrast-enhanced chest–abdomen CT, and periodic dermatologic assessment. At the most recent documented assessment (24 months after surgery), the patient was alive and had no evidence of disease (NED).

## Discussion and conclusion

In large registries, most melanomas arise in the skin, whereas mucosal primaries account for a small fraction (1, 2). When melanoma is identified in the GI tract, metastasis from a cutaneous primary is the most common scenario. Autopsy data and institutional series indicate that GI metastases can be present in a substantial proportion of patients with melanoma, with the small bowel frequently involved (3). Clinically, however, GI involvement is often underdiagnosed because symptoms are nonspecific and small-bowel endoscopic access is limited (3, 7). The concept of a true primary small-bowel melanoma remains controversial, largely because of the possibility of an occult or regressed primary melanoma elsewhere (3–6). Accordingly, most authors recommend treating intestinal melanoma as metastatic until a comprehensive evaluation excludes a primary at more typical sites (skin, eye, and other mucosal locations) and excludes disseminated disease on staging imaging (2, 7). Recent reviews continue to revisit pathogenetic hypotheses, diagnostic criteria, and management considerations for primary gastrointestinal melanoma (8). Several clinicopathologic criteria have been proposed to support a diagnosis of primary small-bowel melanoma. A commonly cited framework includes: (i) a solitary intestinal lesion; (ii) the absence of a cutaneous or other mucosal primary melanoma after thorough examination; and (iii) pathological features suggestive of mucosal origin when present such as an intramucosal *in situ* component or additional intramucosal melanocytic lesions in the adjacent epithelium (often referred to as junctional activity) (2, 6). Importantly, the absence of junctional activity does not exclude a primary lesion, particularly in ulcerated tumors or limited sampling, and should be explicitly reported as in the present case (2) (**Table 1**).

**Table 1:** Practical elements supporting a diagnosis of primary small-bowel melanoma and corresponding findings in the present case [adapted from published criteria (2, 4)].

Element	Rationale	Present case
Solitary intestinal lesion	Primary lesions are typically solitary; multifocal disease is associated with metastasis (2, 4).	Yes (single ileal mass on CT; single lesion resected)
No primary melanoma identified elsewhere.	Exclusion of cutaneous, ocular, and other mucosal primaries is essential (4, 6).	Yes (dermatologic and ophthalmologic evaluations negative)
No disseminated disease on staging	Whole-body staging reduces the risk of misclassifying metastatic disease as primary (4–6).	Yes (CT chest, FDG PET/CT, brain MRI negative)
Regional lymph nodes	Nodal involvement may occur in both settings; documentation is important (4, 6).	Negative (resected nodes negative)
Junctional activity / in situ component	Supports mucosal origin when present; may be absent in ulcerated lesions (4).	Not identified
Sustained disease-free follow-up	Supports (but does not prove) a primary origin when prolonged; a ≥12-month disease-free interval has been proposed as a supportive criterion (2).	Yes (24-month NED)

CT is often the first-line modality in patients presenting with overt GI bleeding or acute anemia, as it can localize the bleeding site, depict an underlying lesion, and evaluate for complications such as obstruction or intussusception. Metastatic melanoma to the small bowel has been described with variable morphologic patterns, including polypoid endoluminal masses, focal or segmental mural thickening, aneurysmal dilatation, and exoenteric growth; lesions may be pigmented or amelanotic (2, 7, 9). In the present case, the key CT features were a hypervascular polypoid ileal mass with marked arterial enhancement and intraluminal hyperdensity consistent with recent hemorrhage, supporting a hypervascular neoplasm as the source of bleeding (**Table 2**).

**Table 2:** Radiologic differential diagnosis for a hypervascular small-bowel mass with bleeding [melanoma-related patterns and staging considerations supported by (3, 4, 6, 8)].

Entity	Typical imaging/pathology clues (non-exhaustive)
Metastatic melanoma	Often polypoid lesions; may be multiple; variable pigmentation; FDG-avid on PET/CT; melanocytic markers positive (S100/SOX10/HMB45/Melan-A) (3, 4, 6, 8).
Primary small-bowel melanoma (probable)	Solitary lesion; no primary elsewhere on thorough evaluation; may show in situ/junctional component (when present) (2, 4).
GIST	Exophytic mass common; enhancement variable; may ulcerate/bleed; IHC typically CD117/DOG1 positive.
Neuroendocrine tumor	Hyperenhancing small lesion; desmoplastic mesenteric reaction; may have calcifications; somatostatin receptor imaging positive.
Adenocarcinoma	Annular constricting lesion; shouldered edges; less marked arterial hyperenhancement; CK positive.
Lymphoma	Segmental thickening, aneurysmal dilatation; bulky nodes; typically, homogeneous and less hypervascular; lymphoid markers positive.

From a radiologic differential diagnosis perspective, a hyperenhancing small-bowel mass causing bleeding includes gastrointestinal stromal tumor (GIST), neuroendocrine tumor, adenocarcinoma, lymphoma (typically less hypervascular), and metastases from hypervascular primaries (e.g., melanoma, renal cell carcinoma). Definitive diagnosis requires histopathology and immunohistochemistry.

Upper endoscopy and colonoscopy may be unrevealing in small-bowel disease. Capsule endoscopy and deep enteroscopy have expanded access to the small intestine and can provide direct visualization and tissue sampling when clinically feasible (7, 10). Enteroscopy can be particularly valuable for confirming the diagnosis, localizing lesions, and guiding surgical planning; however, in the setting of active bleeding or high-risk lesions, cross-sectional imaging often remains the definitive modality for prompt intervention (10, 11). Capsule endoscopy and deep enteroscopy were not pursued because the patient presented with ongoing overt gastrointestinal bleeding associated with severe anemia requiring blood transfusion. Multiphasic CT had already localized the bleeding source and demonstrated a surgically resectable lesion, making additional endoscopic investigation unlikely to alter immediate management and potentially delaying definitive treatment.

Small-bowel melanoma can histologically mimic other neoplasms, particularly when amelanotic. Accordingly, immunohistochemistry is essential and typically relies on a panel of melanocytic markers (e.g., S100, SOX10, HMB45, Melan-A/MART-1), together with exclusion markers tailored to the differential diagnosis (2, 7). In the present case, diffuse expression of S100 and SOX10, positivity for HMB45 and Melan-A, and negativity for cytokeratin excluded carcinoma, while negativity for CD117/DOG1 argued against GIST.

Surgical resection remains the cornerstone for symptomatic small-bowel melanoma, particularly in bleeding or obstructing tumors, and may be considered even in selected metastatic settings for symptom control (3, 7). Population-based analyses of primary gastrointestinal melanoma suggest that outcomes are stage-dependent and that surgical management is associated with improved survival in localized disease, supporting an aggressive resectional approach when feasible (12, 13). Because evidence for adjuvant systemic therapy in resected, localized primary GI melanoma is limited and extrapolated from other melanoma subtypes, postoperative management is often individualized in multidisciplinary discussion (7). In our patient, the absence of residual disease and the negative nodal status supported a surveillance strategy.

The major strengths of this report include: (i) radiologic documentation of a bleeding hypervascular ileal lesion on multiphasic CT; (ii) complete surgical resection with negative margins and negative regional nodes; (iii) comprehensive postoperative staging with CT chest, FDG PET/CT, and contrast-enhanced brain MRI; and (iv) a 24-month disease-free follow-up.

Limitations include the inherent impossibility of definitively excluding a regressed occult primary melanoma and the lack of an identified junctional/*in situ* component on pathology. Recent short communications and case reports continue to emphasize these diagnostic pitfalls and the frequency of bleeding presentations in intestinal melanoma, whether primary or metastatic (14, 15). Nevertheless, when interpreted together, the solitary lesion, negative whole-body staging, and sustained NED follow-up support classification as a probable primary ileal melanoma (2, 7).

Ileal melanoma presenting with active small-bowel bleeding is rare and poses an immediate diagnostic challenge. Multiphasic contrast-enhanced CT is pivotal for detecting the culprit lesion and signs of hemorrhage, while whole-body FDG PET/CT provides critical staging information. In the present node-negative patient, the absence of a detectable cutaneous or ocular primary and the lack of disseminated disease on staging, together with sustained disease-free follow-up, support the diagnosis of probable primary small-bowel melanoma. Careful multidisciplinary assessment, transparent reporting of pathology (including the presence/absence of junctional activity), and long-term follow-up are essential to strengthen classification and guide management.

## Data Availability

The original contributions presented in the study are included in the article/Supplementary Material. Further inquiries can be directed to the corresponding author.
